# Spatiotemporal Analysis of Serogroup C Meningococcal Meningitis Spread in Niger and Nigeria and Implications for Epidemic Response

**DOI:** 10.1093/infdis/jiz343

**Published:** 2019-10-31

**Authors:** Laura V Cooper, Olivier Ronveaux, Katya Fernandez, Clement Lingani, Kadade Goumbi, Chikwe Ihekweazu, Marie-Pierre Preziosi, Antoine Durupt, Caroline L Trotter

**Affiliations:** 1 University of Cambridge, Cambridge, United Kingdom, Geneva, Switzerland; 2 Department of Pandemic and Epidemic Diseases, World Health Organization, Geneva, Switzerland; 3 Inter-country Support Team for West Africa, World Health Organization, Ouagadougou, Burkina Faso; 4 Ministry of Public Health, Niamey, Niger; 5 Nigeria Center for Disease Control, Abuja, Nigeria; 6 Department of Immunization, Vaccines and Biologicals, World Health Organization, Geneva, Switzerland

**Keywords:** epidemic response, meningitis, Niger, Nigeria, vaccine

## Abstract

**Background:**

After the re-emergence of serogroup C meningococcal meningitis (MM) in Nigeria and Niger, we aimed to re-evaluate the vaccination policy used to respond to outbreaks of MM in the African meningitis belt by investigating alternative strategies using a lower incidence threshold and information about neighboring districts.

**Methods:**

We used data on suspected and laboratory-confirmed cases in Niger and Nigeria from 2013 to 2017. We calculated global and local Moran’s I-statistics to identify spatial clustering of districts with high MM incidence. We used a Pinner model to estimate the impact of vaccination campaigns occurring between 2015 and 2017 and to evaluate the impact of 3 alternative district-level vaccination strategies, compared with that currently used.

**Results:**

We found significant clustering of high incidence districts in every year, with local clusters around Tambuwal, Nigeria in 2013 and 2014, Niamey, Niger in 2016, and in Sokoto and Zamfara States in Nigeria in 2017.

We estimate that the vaccination campaigns implemented in 2015, 2016, and 2017 prevented 6% of MM cases. Using the current strategy but with high coverage (85%) and timely distribution (4 weeks), these campaigns could have prevented 10% of cases. This strategy required the fewest doses of vaccine to prevent a case. None of the alternative strategies we evaluated were more efficient, but they would have prevented the occurrence of more cases overall.

**Conclusions:**

Although we observed significant spatial clustering in MM in Nigeria and Niger between 2013 and 2017, there is no strong evidence to support a change in methods for epidemic response in terms of lowering the intervention threshold or targeting neighboring districts for reactive vaccination.

Since guidelines were first issued by the World Health Organization (WHO) in 1995, reactive vaccination policy in the African meningitis belt has evolved in response to changes in disease burden and continuing insights from research. The first set of guidelines proposed an incidence threshold indicating high epidemic risk at 15 or more suspected cases of meningitis per 100 000 population per week over a period of 2 weeks [1]. This was updated in 2000 to recommend a lower threshold of 10 suspected cases per 100 000 per week for high-risk districts and to emphasize the importance of surveillance at a district level, because outbreaks of meningitis tend to occur at a fine spatial scale and can be missed when surveillance is carried out more coarsely [2]. A recommendation was also made for vaccination of districts in alert (exceeding incidence of 5 suspected cases per 100 000 per week) and neighboring an epidemic district. With the introduction of group A meningococcal conjugate vaccine in the meningitis belt in 2010 and subsequent reduction in the burden of group A meningococcal meningitis, these thresholds were re-evaluated, focusing on *Neisseria meningitidis* (Nm) serogroup W outbreaks [3]. This research informed the next iteration of WHO guidelines, which maintained the epidemic threshold of 10 suspected cases per 100 000 per week but emphasized the importance of surveillance in populations smaller than 100 000 persons and minimizing delay between triggering of the incidence threshold and intervention. The guidelines also lowered the alert threshold to 3 suspected cases per 100 000 per week and relaxed the recommendation for vaccination in neighboring districts to allow for more flexibility [4].

Prior studies of meningitis patterns in Niger have demonstrated significant spatial heterogeneity and strong interannual and intradistrict variation in meningitis incidence, and authors have suggested that surveillance on finer spatial scales may improve the timeliness and targeting of epidemic response [5–7]. In particular, these studies find that performing outbreak detection on the subdistrict level and response on the district level is more efficient than traditional detection at the district level. In light of the recent emergence and continued epidemics of serogroup C meningococcal meningitis in Nigeria and Niger, we aimed to re-evaluate the reactive vaccination policy used to respond to seasonal outbreaks of meningococcal meningitis in the African meningitis belt and investigated alternative strategies. Although subdistrict-level data are not widely available, we wanted to investigate whether targeting neighbors at the district level might allow for more efficient use of vaccine. We also wanted to extend the scope of these earlier studies by including data from Nigeria in our analysis, and considering the full time period from the emergence of the outbreak strain in 2013 [8].

## METHODS

### Surveillance Data

We used surveillance data on suspected cases of meningitis from the enhanced district-level surveillance system established in 2003 [9]. A suspected case is defined as any person with sudden onset of fever (>38.5°C rectal or 38.0°C axillary) and any one of the following signs: neck stiffness, flaccid neck (infants), bulging fontanelle (infants), convulsion, or other meningeal signs. Weekly district-level counts of suspected cases are collated nationally and then reported to the WHO Inter-Country Support Team (WHO-IST) for West Africa. We used data from Niger and Nigeria during the period from 2013 to 2017, when serogroup C was dominant. We used the district-level population sizes reported by each country to WHO-IST, which are updated annually.

We used national-level annual data on confirmed cases of meningitis to estimate the overall proportion of cases due to serogroup C and preventable by vaccination (Table 1). Cerebrospinal fluid (CSF) samples are tested by national reference laboratories using polymerase chain reaction, culture, or latex agglutination, and these results are collated by WHO-IST [10]. Because of the lack of microbiological data for Nigeria in 2016, we assumed the etiological proportions were the same as in Niger 2016 for the purposes of the model. We also used anonymized line list data, which includes the age of cases and more detailed laboratory confirmation data for Niger 2015 to 2017 and Nigeria 2017, to evaluate the accuracy of some of our model assumptions.

**Table 1.** Confirmed and Suspected Cases of Meningitis and Estimated Proportion of Suspected Cases That Could Have Been Prevented by C, ACW, and ACWY Vaccines Between 2013 and 2017 in Niger and Nigeria^a^

Year	Country	Suspected Meningitis Cases	Total Confirmed Nm	A	C	X	Y	W	Covered by C	Covered by ACW	Covered by ACWY
2013	Niger	311	11	0	0	0	0	11	0%	100%	100%
	Nigeria	871	10	3	7	0	0	0	70%	100%	100%
2014	Niger	315	24	0	8	0	0	16	33%	100%	100%
	Nigeria	1175	38	0	38	0	0	0	100%	100%	100%
2015	Niger	7978	1436	0	1183	1	0	206	82%	97%	97%
	Nigeria	2655	20	0	20	0	0	0	100%	100%	100%
2016	Niger	1976	357	0	312	15	0	25	87%	94%	94%
	Nigeria	831	…	…	22	…	…	…	…	…	…
2017	Niger	3387	1073	0	848	220	0	4	79%	79%	79%
	Nigeria	9918	18	1	14	0	0	1	78%	89%	89%

Abbreviations: Nm, *Neisseria meningitidis*.

^a^A, C, X, Y, W columns may not add up to total confirmed Nm due to nontypeable isolates.

### Maps

We matched districts to their locations on district-level maps obtained from WHO using place names. Where no match was found between the map names and a surveillance database place name, we used the GeoNames database to situate the unknown place name within a known district [11]. Large districts that divided into 2 or more smaller districts during the study period (2013–2017) were kept as a single district to allow for tracking of vaccine coverage over time and to avoid introducing bias by sudden population changes (Supplementary Table S1). We defined neighbors as those districts sharing a border with the focal district. This definition of neighboring districts included those across national borders.

### Cluster Detection

We tested for nonrandom spatial distribution of annual cumulative incidence using the Global Moran’s I statistic for each year from 2013 to 2017 [12]. Measures of spatial autocorrelation can be highly dependent on the imposed spatial structure, ie, what is considered a neighbor and how each neighbor is weighted [13]. For this reason, we calculated Moran’s global I statistic in R using the spdep package with 3 different weighting structures—by simple contiguity, great circle distance (within 50 km of centroid), and taking the 5 nearest neighbors, with distance again calculated by great circle centroid-to-centroid (Supplementary Table S2). For the distance-based weight, we evaluated distances between 20 and 100 kilometers. Fifty kilometers was chosen because it maximized spatial effects in most years. All districts meeting neighbor criterion were weighted equally. The significance of these values was determined by rank within 999 Monte Carlo permutations.

**Figure 1.**
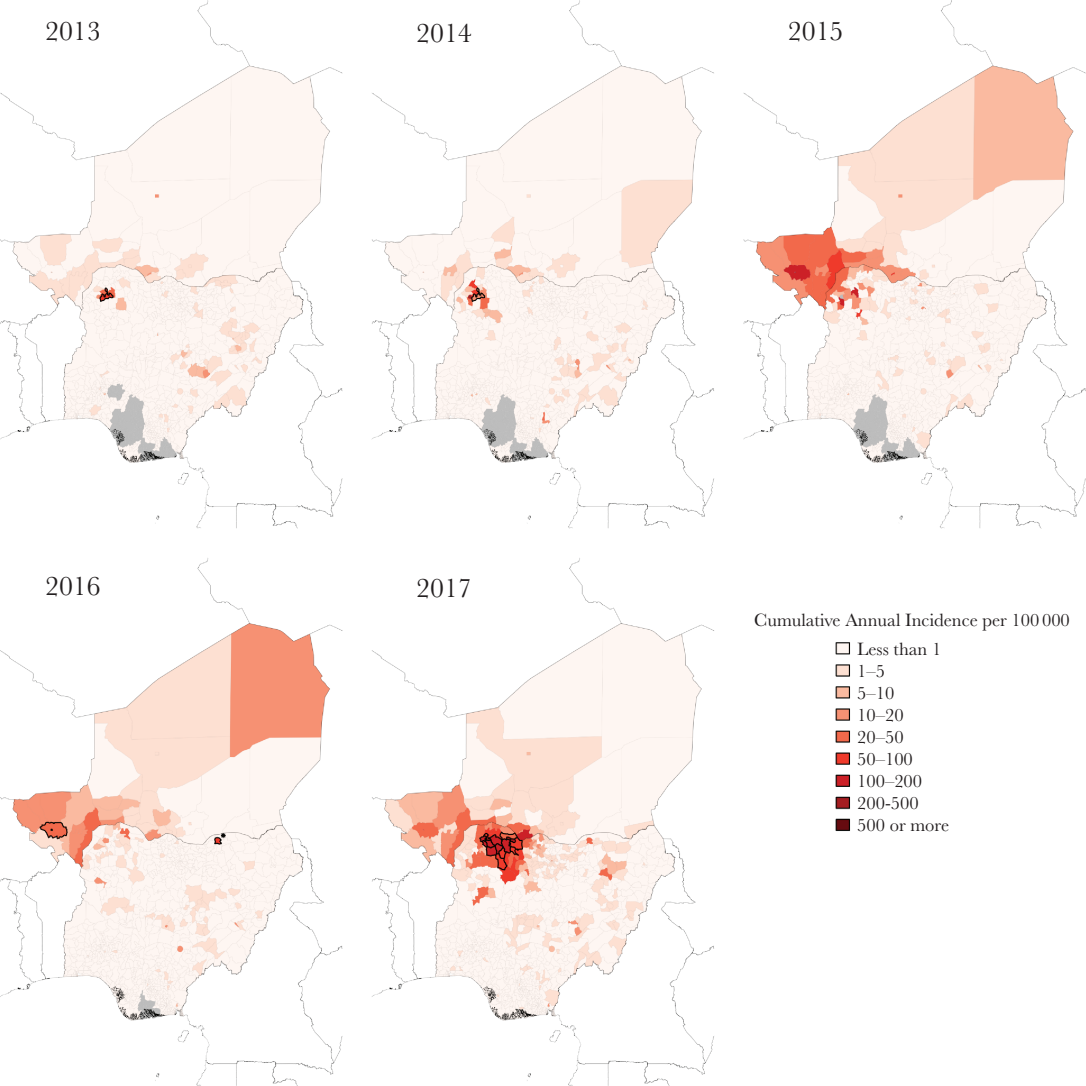
Cumulative annual district-level incidence of suspected cases of meningitis in Niger and Nigeria 2013 to 2017. Centers of significant clusters of high incidence by Anselin’s local Moran’s I outlined in black. High-incidence outlier in 2016 indicated with asterisk. Gray areas represent districts for which no data are reported.

Because all weighting methods detected significant positive spatial autocorrelation of similar magnitude in each year, we continued by using only contiguity weights for our analysis of local clusters. We calculated Anselin’s local Moran’s I to locate clusters of districts with high incidence or outlier districts with higher incidence than neighbors [5, 14]. We used the spdep package in R and GeoDa software to calculate local Moran’s I statistics for each district in each year. In GeoDa, we used a permutation approach with 99 999 permutations to generate pseudo *P* values. We then compared these to the analytical results from the spdep packages. We used an overall alpha of 0.05 and a Bonferroni adjustment for repeated testing. Because the analytical *P* values and the pseudo *P* values gave inconsistent results, we reported only the clusters found significant by both methods (Supplementary Table S3, Figure 1).

### Definitions

We refer to the raw data of weekly suspected cases as observed suspected cases (OSCs), and we refer to cases expected in the absence of the vaccination campaigns taking place in selected districts in 2015, 2016, and 2017 as modeled suspected cases. Epidemic and alert incidence thresholds are consistent with current WHO guidelines, with alert defined as more than 3 suspected cases per 100 000 population per week or more than 2 cases total per week for districts with less than 30 000 population and epidemic with as more than 10 suspected cases per 100 000 population per week or more than 5 cases total per week or a doubling of cases in a 2-week period for districts with less than 30 000 population [4]. We define a district-level meningitis outbreak period where Nm is the dominant cause of meningitis as the period in which incidence of OSCs exceeds the alert incidence threshold.

### Estimating Cases in the Absence of Intervention

Reactive vaccination campaigns occurred in 2015, 2016, and 2017 in 56 districts in response to NmC outbreaks using polysaccharide vaccine, monovalent C conjugate vaccine, and quadrivalent ACWY conjugate vaccine. To model suspected cases of meningitis in the absence of vaccination, we made the following assumptions: (1) 80% of suspected cases during the outbreak period are due to Nm; (2) 20% of cases outside the outbreak period are due to Nm; (3) the serogroup distribution is proportional to national annual proportions; (4) there is a delay of 6 weeks between triggering of the epidemic threshold and protection from vaccination (Table 2). Where no threshold was triggered but vaccination still took place, we made a best guess for the timing of the intervention based on neighboring districts. For polysaccharide vaccine, we further assumed the following: (1) campaigns target 2- to 29-year-olds, who make up 70% of the population and 90% of cases [15, 16]; (2) 80% vaccine efficacy against serogroups A, C, and W [17]; and (3) protection lasts for 104 weeks (approximately 2 years) [18]. This type of model has been used by many authors in the past to estimate the impact of reactive vaccination for meningitis outbreaks [3, 19, 20]. More importantly, it assumes no impact of the vaccine on carriage and thus transmission of meningococci.

**Table 2.** Summary of Model Assumptions

Model	Coverage	Vaccine Efficacy	Serogroups Covered	Duration of Protection	Delay	Herd Effects	Population Targeted
Polysaccharide vaccination (2015–2017)	Calculated from ICG data	80%	A, C, W	2 years	6 weeks	No	2–29 years; 70% of population
Monovalent conjugate C vaccination (2017)	Calculated from ICG data	85%	C	5 years (average)	6 weeks	Yes	1–19 years
Quadrivalent conjugate vaccination (2015)	Calculated from ICG data	85%	A, C, W, Y	5 years (average)	6 weeks	Yes	2–15 years
Theoretical polysaccharide vaccination	85%	80%	A, C, W	2 years	4–8 weeks	No	2–29 years; 70% of population

Abbreviations: ICG, International Coordinating Group.

For the monovalent C conjugate vaccine, we used output from a dynamic model to approximate herd effects, assuming 85% vaccine efficacy against serogroup C meningitis, an average of 5 years duration of protection, and coverage levels calculated from reported population size and vaccine use [21, 22]. For the quadrivalent conjugate vaccine, we used output from the same dynamic model to approximate herd effects, assuming 98% coverage of 2- to 15-year-olds (reflecting the actual coverage in Ouallam), 85% vaccine efficacy against serogroups A, C, W, and Y, and an average 5 years duration of protection [23].

Vaccine coverage is calculated using the district-level population reported in surveillance data, assuming the targeted population is 2- to 29-year-olds, representing 70% of total population, and the number of doses of vaccine released by the International Coordinating Group on Vaccine Provision or population vaccinated as reported in the Weekly Epidemiological Record where available [24–26]. For all coverage calculations, we assumed 10% vaccine wastage.

### Modeling New Vaccination Strategies

We then modeled uniform application of the current strategy and 3 alternative reactive vaccination strategies, making the same assumptions as described above (Table 2). Under the current strategy, mass vaccination takes place on a district level where the weekly incidence of suspected cases exceeds the epidemic threshold and the causative serogroup can be identified. We drop this etiological confirmation requirement for modeling because weekly district-level laboratory data were not available. In alternative strategy A, vaccination takes place when the alert threshold is exceeded. In strategy B, all neighboring districts that have not received vaccine in the last 5 or 2 years for conjugate and polysaccharide vaccine, respectively, are targeted in addition to the district exceeding the epidemic threshold. In strategy C, only those neighboring districts in alert are targeted in addition to the focal district in epidemic. To compare these strategies, we modeled use of a trivalent polysaccharide vaccine, making the following assumptions: (1) campaigns target 2- to 29-year-olds with 85% coverage, who make up 70% of the population and 90% of cases [15, 16]; (2) 80% vaccine efficacy against serogroups A, C, and W [17]; (3) protection lasts for 104 weeks (approximately 2 years) [18]. We model delays between triggering a vaccine response and onset of vaccine protection as 4, 6, and 8 weeks, effectively giving 2, 4, and 6 weeks for implementation and 2 weeks for vaccine protection to take effect after delivery [17].

### Analysis of Model Results

We evaluated the performance of the different strategies and vaccine types by calculating the cases averted, doses of vaccine required to prevent a case (NNV), and the sensitivity, specificity, positive predictive value (PPV), and negative predictive value (NPV) of each strategy for predicting outbreaks defined by different cumulative annual incidences (from 100 cases per 100 000 to 20 cases per 100 000).

## RESULTS

### Spatial and Temporal Characterization

**Figure 2.**
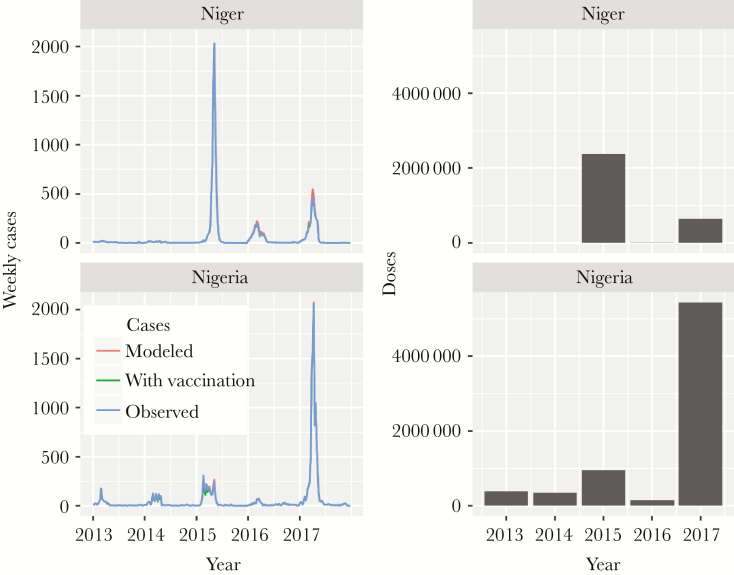
Observed and modeled weekly case counts, annual cases averted by reactive vaccination, and doses vaccine delivered in Niger and Nigeria 2013 to 2017.

Outbreak activity was first observed in northern Nigeria in 2013 and continued in 2014 (Figures 1 and 2). In 2015, activity spread to the southwestern region of Niger, including the urban area of Niamey. Fewer outbreaks were observed in 2016, followed by relatively high case counts in Niger and the highest case counts in Nigeria thus far in 2017. We detected 20 district-years with high incidence and positive spatial autocorrelation (ie, centers of clusters of high incidence), 46 districts neighboring these clusters, and 1 district with high incidence and negative spatial autocorrelation, meaning it was an outlier from its neighbors). Tambuwal LGA in Sokoto State in Nigeria, where the group C outbreak strain was first identified, was detected as the center of high incidence clusters in 2013 and 2014, but it did not pass the weekly epidemic incidence threshold (WIT10) of 10 cases per 100 000 in 2014 and did not exceed a cumulative annual incidence of 100 suspected cases per 100 000 in either year (CAI100) [8]. Of the neighboring districts, 2 and 4 districts, respectively surpassed the WIT10 in 2013 and 2014 and none exceeded the CAI100. No significant clusters were detected in 2015. High incidence clustered around Niamey in Niger in 2016. Only 1 district, Niamey I, exceeded the WIT10, and none exceeded the CAI100 (although Niamey I came close with 96 cases per 100 000). There was also a spatial outlier of high incidence identified in Nguru LGA in Yobe State in north-central Nigeria. In 2017, 2 large clusters were detected in Sokoto State and Zamfara State in Nigeria. All 15 of the center districts and 16 of the 21 neighboring districts surpassed WIT10 between weeks 10 and 18. Nine of the center districts in these clusters and 2 neighboring districts exceeded the CAI100. Supplementary Table S3 gives full details of all clusters detected.

### Impact of Reactive Campaigns

Reactive vaccination campaigns were conducted in 2015, 2016, and 2017 in 60 districts in response to NmC outbreaks using polysaccharide vaccine, monovalent C conjugate vaccine, and quadrivalent ACWY conjugate vaccine (Supplementary Table S4). Our model estimates that these campaigns prevented 1100 of 19 000 (6%) cases of meningococcal meningitis overall during the period 2015 to 2017 (270 of 10 000 [3%] cases in Nigeria and 830 of 9000 [9%] cases in Niger).

### Modeling Alternative Reactive Vaccination Strategies

**Figure 3.**
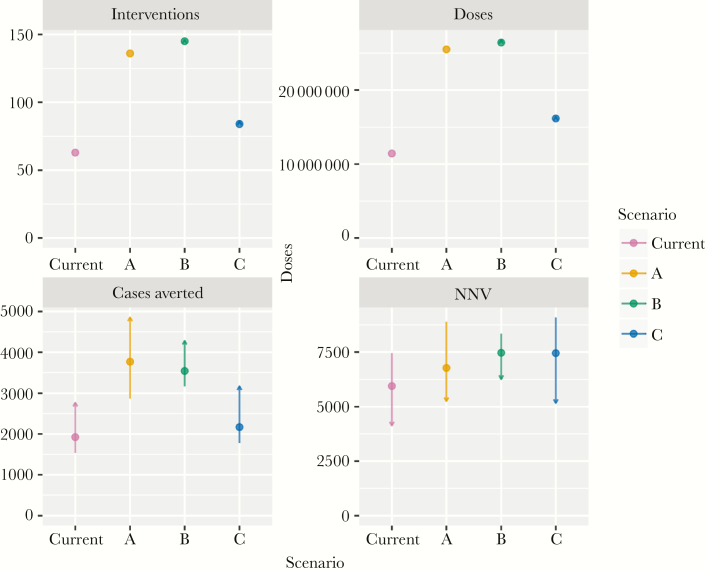
Number of interventions (individual districts vaccinated), doses, proportion of total cases averted, and number needed to vaccinate to prevent a case over the period 2013–2017 for different reactive vaccination strategies using polysaccharide ACW vaccine. Points show estimates for 4-week delays, lines show 2- and 6-week delays, with arrow heads indicating shorter delays.

The current strategy required fewest interventions with 63 districts requiring 11 million doses of polysaccharide vaccine between 2013 and 2017, followed by alternative strategy C, which would have targeted an additional 21 districts with 5 million additional doses (Figure 3). Strategies A and B would have required targeting 73 and 82 additional districts, respectively, requiring an additional 14 and 15 million doses of polysaccharide vaccine.

**Figure 4.**
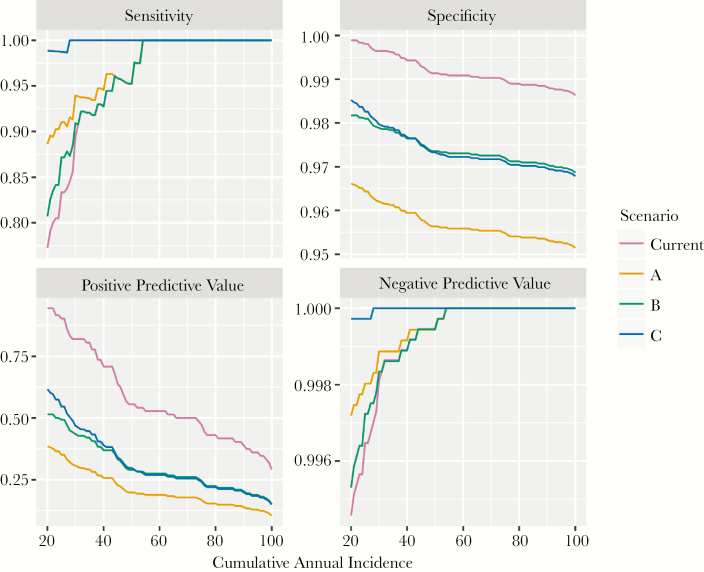
Sensitivity, specificity, positive predictive value, and negative predictive value of different targeting strategies for predicting cumulative annual incidence between 20 and 100 suspected cases per 100 000.

Strategy C had the greatest sensitivity in predicting small outbreaks, whereas the current strategy had the least (Figure 4). Sensitivity was comparable across all strategies for large outbreaks. The current strategy had the highest PPV at any cumulative incidence threshold, followed by strategy C. Specificity and NPV were similar across all strategies. Strategies A and B prevented the most cases, 19% and 18% of all meningococcal meningitis cases, followed by strategy C (11%) and the current strategy (10%).

With shorter delays of 2 weeks, A prevented the most cases (25%); with longer delays of 6 weeks, B prevented the most cases (16%). Across all strategies, interventions with delays of 2 weeks prevented between 1.4 and 1.8 times as many cases those with delays of 6 weeks. Shortening the delay by 2 weeks while maintaining the current strategy would have prevented 14% of cases, close to the proportion prevented by strategies A and B while requiring no additional vaccine. No strategy outperformed the current strategy in terms of NNV, but A was the second most efficient use of vaccine, with an NNV of 6800 compared with the current strategy at 5900.

## DISCUSSION

We found significant clustering of high incidence districts in every year, with local clusters occurring around Tambuwal LGA in Nigeria in 2013 and 2014, around Niamey, Niger in 2016, and 2 around Sokoto and Zamfara States in Nigeria in 2017. The locations of these local clusters are consistent with areas of elevated incidence. No significant local cluster was identified in 2015 although incidence was high in Niamey and Tillaberi Regions, perhaps because the distribution of incidence was more even in space.

Our modeling analyses provide no strong evidence to support a change in the methods currently used for predicting and targeting outbreaks. The current weekly incidence threshold of 10 suspected cases per 100 000 is highly specific and sensitive to large outbreaks. Shortening delays between outbreak detection and vaccination offers a substantial benefit. These 2 results are consistent with previous findings regarding group W outbreak response [3]. Although the absolute values in NNV may differ slightly between this and previous studies, adjustment for differing assumptions about coverage and vaccine efficacy can largely account for these differences [6, 27]. However, we note that the majority of districts were larger than 100 000 population, which is not consistent with WHO recommendations. Previous studies have demonstrated that surveillance at finer spatial scales may allow for more efficient and earlier use of vaccine where it is needed [7, 28].

This study also highlights the substantial difference between the number of doses of vaccine required to intervene as per WHO policy (approximately 11 million) and the number of doses that were made available and used in reactive vaccination campaigns between 2013 and 2017 (approximately 4 million) [29]. Although some of this shortfall can be explained by the necessity of dropping the etiological confirmation requirement in our model, it is clear that vaccine scarcity had an impact on decisions about where to carry out reactive vaccination and for which age groups. Although data from Nigeria on laboratory-confirmed cases of meningitis were lacking, one study confirmed our assumption that NmC was the predominant cause of meningitis in Nigeria, with 172 of 173 Nm isolates being from serogroup C [30].

Informed by expert opinion, our model assumes that 80% of suspected meningitis cases occurring during outbreaks are caused by Nm, with just 20% of suspected cases caused by Nm outside of outbreak periods. Line list data from Niger between 2015 and 2017 and Nigeria in 2015 suggests that only 27% of suspected cases occurring during outbreaks are confirmed as meningococcal meningitis, with a similar proportion occurring outside of outbreak periods (Supplementary Table S5). A majority of CSFs test negative for common bacterial pathogens both during and outside of outbreak periods. It is possible that this is driven by antibiotic administration before taking CSF, sample degradation, and the low sensitivity of latex agglutination tests and culture methods compared with polymerase chain reaction-based methods [31–34]. However, we cannot exclude the possibility that there may be substantial clinical misclassification of suspected meningitis cases.

## CONCLUSIONS

The epidemiological behavior of meningococcal meningitis in the African meningitis belt is notoriously difficult to predict. Our results will only apply to future epidemiological situations insofar as the spatiotemporal distribution and magnitude of group C meningitis outbreaks remain approximately the same. There is a high degree of uncertainty about which epidemiological characteristics may be considered typical given the strain’s recent emergence. In any case, it is important to maintain high-quality surveillance throughout the meningitis belt, striving especially to improve laboratory diagnostics in outbreak settings.

## Supplementary Data

Supplementary materials are available at *The Journal of Infectious Diseases* online. Consisting of data provided by the authors to benefit the reader, the posted materials are not copyedited and are the sole responsibility of the authors, so questions or comments should be addressed to the corresponding author.

jiz343_suppl_Supplementary_Material

## Notes

***Financial support.*** This work was funded by the MenAfriNet consortium (www.menafrinet.org) through a grant from the Bill & Melinda Gates Foundation (OPP1084298).

***Potential conflicts of interest.*** All authors: No reported conflicts of interest. All authors have submitted the ICMJE Form for Disclosure of Potential Conflicts of Interest.

Presented in part: 5th Annual Meeting on Surveillance, Preparedness and Response to Meningitis Outbreaks in Africa and Meeting of MenAfriNet Partners, October 2018, Dakar, Senegal.
