# Supplementary material for: Spatiotemporal Analysis of Serogroup C Meningococcal Meningitis Spread in Niger and Nigeria and Implications for Epidemic Response
Source: J Infect Dis. 2019 Oct 31;220(Suppl 4):S244–52. doi: 10.1093/infdis/jiz343 (PMC6822969; doi:10.1093/infdis/jiz343)
Supplement: jiz343_suppl_Supplementary_Material [file jiz343_suppl_supplementary_material.docx]

Supplementary Information

**Table S1.** District names in surveillance and map data sets.

| Country | Region | District (surveillance) | District (map) | Year |
| --- | --- | --- | --- | --- |
| Niger | Agadez | Aderbissanat | Tchirozerine | 2016-2017 |
| Niger | Agadez | Iferouane | Arlit | 2017 |
| Niger | Agadez | Ingall | Tchirozerine | 2017 |
| Niger | Diffa | Bosso | Diffa | 2017 |
| Niger | Diffa | Goudoumaria | Maine Soroa | 2017 |
| Niger | Diffa | N'gourti | N'guigmi | 2017 |
| Niger | Dosso | Dioundiou | Gaya | 2016-2017 |
| Niger | Dosso | Falmeye | Boboye | 2017 |
| Niger | Dosso | Tibiri | Guidam-roumdji | 2016-2017 |
| Niger | Maradi | Bermo | Dakoro | 2017 |
| Niger | Maradi | Gazaoua | Tessaoua | 2017 |
| Niger | Maradi | Guidan-Roumdji | Guidam-roumdji | 2013-2017 |
| Niger | Niamey | Niamey II | Niamey I | 2015-2017 |
| Niger | Niamey | Niamey III | Niamey II | 2015-2017 |
| Niger | Niamey | Niamey IV | Niamey II | 2015-2017 |
| Niger | Niamey | Niamey V | Niamey III | 2015-2017 |
| Niger | Tahoua | Bagaroua | Illela | 2017 |
| Niger | Tahoua | Malbaza | Birni N'konni | 2016-2017 |
| Niger | Tahoua | Tahoua Commune | Tahoua | 2017 |
| Niger | Tahoua | Tahoua Dept | Tahoua | 2016-2017 |
| Niger | Tahoua | Tassara | Tchintabaraden | 2017 |
| Niger | Tahoua | Tillia | Tchintabaraden | 2017 |
| Niger | Tillaberi | Abala | Filingue | 2017 |
| Niger | Tillaberi | Ayorou | Tera | 2017 |
| Niger | Tillaberi | Balleyara | Filingue | 2017 |
| Niger | Tillaberi | Banibangou | Ouallam | 2016-2017 |
| Niger | Tillaberi | Bankilare | Tera | 2017 |
| Niger | Tillaberi | Gotheye | Tillaberi | 2017 |
| Niger | Tillaberi | Torodi | Say | 2017 |
| Niger | Zinder | Belbedji | Tanout | 2017 |
| Niger | Zinder | Damagaram T | Mirriah | 2017 |
| Niger | Zinder | Takeita | Mirriah | 2017 |
| Nigeria | Adamawa | Lamurde | Larmurde | 2013-2015 |
| Nigeria | Adamawa | Toungo | Teungo | 2013-2015 |
| Nigeria | Benue | Buruku | Bukuru | 2013 |
| Nigeria | Benue | Makurdi | Markurdi | 2013-2017 |
| Nigeria | Benue | Ogbdibo | Ogbadibo | 2013 |
| Nigeria | Benue | Otukpo | Oturkpo | 2013 |
| Nigeria | Gombe | Shongom | Shomgom | 2013-2015 |
| Nigeria | Imo | Ezinihitte Mbaise | Ezinihitte | 2013-2015 |
| Nigeria | Imo | Mbaitoli | Mbatoli | 2013-2015 |
| Nigeria | Imo | Onuimo | Unuimo | 2013-2015 |
| Nigeria | Jigawa | Birniwa | Biriniwa | 2013-2015 |
| Nigeria | Jigawa | Kiri-Kasamma | Kiri Kasama | 2013-2015 |
| Nigeria | Jigawa | Sule-Tankarkar | Sule Tankakar | 2013-2015 |
| Nigeria | Kaduna | Kubau | Kuban | 2013-2015 |
| Nigeria | Kano | Ungogo | Ungongo | 2013-2015 |
| Nigeria | Katsina | Jibya | Jibia | 2013-2015 |
| Nigeria | Katsina | Maiaduwa | Mai'Adua | 2013-2015 |
| Nigeria | Katsina | Malunfashi | Malumfashi | 2013 |
| Nigeria | Kebbi | Aliero | Aleiro | 2013-2015 |
| Nigeria | Kebbi | Arewa | Arewa Dandi | 2013-2015 |
| Nigeria | Kebbi | Danko Wasagu | Wasagu/Danko | 2013-2015 |
| Nigeria | Kebbi | Koko Bese | Koko/Besse | 2013-2015 |
| Nigeria | Kwara | Patigi | Pategi | 2013-2015 |
| Nigeria | Nasarawa | N/Eggon | Nasarawa Egon | 2013-2015 |
| Nigeria | Niger | Minna | Chanchaga | 2013-2015 |
| Nigeria | Niger | Munya | Muya | 2013-2015 |
| Nigeria | Oyo | Ogbomoso North | Ogbomosho North | 2013-2015 |
| Nigeria | Oyo | Ogbomoso South | Ogbomosho South | 2013-2015 |
| Nigeria | Oyo | Oorelope | Orelope | 2013-2015 |
| Nigeria | Taraba | K/Lamido | Karim Lamido | 2013 |
| Nigeria | Yobe | Bade | Barde | 2013 |
| Nigeria | Yobe | Bursari | Borsari | 2013-2015 |
| Nigeria | Yobe | Tarmuwa | Tarmua | 2013-2015 |
| Nigeria | Zamfara | Birnin Magaji | Birnin Magaji/Kiyaw | 2013-2015 |
| Nigeria | Zamfara | Birnin Magaji/Kiyawa | Birnin Magaji/Kiyaw | 2016-2017 |

**Table S2.** Moran’s global I statistic for each year using three types of spatial weighting structures. P-values from 999 Monte Carlo permutations.

| Year | Moran’s global I statistic | | |
| --- | --- | --- | --- |
|  | Contiguity | Distance (50 km) | Five nearest neighbours |
| 2013 | 0.28 (1e-04) | 0.29 (1e-04) | 0.33 (4e-04) |
| 2014 | 0.19 (1e-04) | 0.16 (1e-04) | 0.15 (9e-04) |
| 2015 | 0.23 (3e-04) | 0.15 (0.0013) | 0.21 (0.0012) |
| 2016 | 0.49 (1e-04) | 0.33 (1e-04) | 0.4 (1e-04) |
| 2017 | 0.69 (1e-04) | 0.7 (1e-04) | 0.68 (1e-04) |

**Table S3.** Significant clusters and outliers of cumulative annual district-level incidence detected by Anselin’s local Moran’s I-statistic.

| Year | District | Region | Country | Cluster centre, cluster or outlier? | Population | Week crossing epidemic threshold | Cumulative annual incidence per 100,000 |
| --- | --- | --- | --- | --- | --- | --- | --- |
| 2013 | Tambuwal | Sokoto | Nigeria | Cluster centre | 276637 | 8 | 87 |
| 2013 | Aliero | Kebbi | Nigeria | Cluster | 81692 |  | 20 |
| 2013 | Argungu | Kebbi | Nigeria | Cluster | 242059 |  | 2 |
| 2013 | Augie | Kebbi | Nigeria | Cluster | 145231 |  | 0 |
| 2013 | Gummi | Zamfara | Nigeria | Cluster | 254996 |  | 1 |
| 2013 | Gwandu | Kebbi | Nigeria | Cluster | 187000 | 9 | 35 |
| 2013 | Jega | Kebbi | Nigeria | Cluster | 239419 |  | 0 |
| 2013 | Kebbe | Sokoto | Nigeria | Cluster | 153314 |  | 0 |
| 2013 | Shagari | Sokoto | Nigeria | Cluster | 192368 | 9 | 46 |
| 2013 | Yabo | Sokoto | Nigeria | Cluster | 141449 |  | 1 |
| 2014 | Tambuwal | Sokoto | Nigeria | Cluster centre | 284936 |  | 15 |
| 2014 | Aliero | Kebbi | Nigeria | Cluster | 84224 | 4 | 261 |
| 2014 | Argungu | Kebbi | Nigeria | Cluster | 249563 |  | 8 |
| 2014 | Augie | Kebbi | Nigeria | Cluster | 149734 | 12 | 55 |
| 2014 | Gummi | Zamfara | Nigeria | Cluster | 263156 |  | 30 |
| 2014 | Gwandu | Kebbi | Nigeria | Cluster | 192797 | 12 | 73 |
| 2014 | Jega | Kebbi | Nigeria | Cluster | 246841 |  | 22 |
| 2014 | Kebbe | Sokoto | Nigeria | Cluster | 157913 |  | 0 |
| 2014 | Shagari | Sokoto | Nigeria | Cluster | 198139 | 8 | 12 |
| 2014 | Yabo | Sokoto | Nigeria | Cluster | 145692 |  | 2 |
| 2016 | Kollo |  | Niger | Cluster centre | 511496 |  | 28 |
| 2016 | Niamey I |  | Niger | Cluster centre | 503655 | 8 | 96 |
| 2016 | Niamey III |  | Niger | Cluster centre | 145801 |  | 23 |
| 2016 | Boboye |  | Niger | Cluster | 385387 |  | 7 |
| 2016 | Filingue |  | Niger | Cluster | 613430 |  | 6 |
| 2016 | Niamey II |  | Niger | Cluster | 482426 |  | 50 |
| 2016 | Ouallam |  | Niger | Cluster | 431800 |  | 16 |
| 2016 | Say |  | Niger | Cluster | 393721 |  | 4 |
| 2016 | Tera |  | Niger | Cluster | 727727 |  | 11 |
| 2016 | Tillaberi |  | Niger | Cluster | 312550 |  | 10 |
| 2016 | Nguru | Yobe | Nigeria | Outlier | 212481 |  | 51 |
| 2017 | Anka | Zamfara | Nigeria | Cluster centre | 201197 | 12 | 65 |
| 2017 | Bakura | Zamfara | Nigeria | Cluster centre | 264300 | 12 | 108 |
| 2017 | Bodinga | Sokoto | Nigeria | Cluster centre | 242803 | 12 | 135 |
| 2017 | Dange-Shuni | Sokoto | Nigeria | Cluster centre | 269297 | 13 | 188 |
| 2017 | Isa | Sokoto | Nigeria | Cluster centre | 202241 | 14 | 35 |
| 2017 | Kaura Namoda | Zamfara | Nigeria | Cluster centre | 397878 | 13 | 97 |
| 2017 | Kware | Sokoto | Nigeria | Cluster centre | 185348 | 17 | 76 |
| 2017 | Maradun | Zamfara | Nigeria | Cluster centre | 298163 | 13 | 116 |
| 2017 | Rabah | Sokoto | Nigeria | Cluster centre | 206479 | 11 | 127 |
| 2017 | Sokoto North | Sokoto | Nigeria | Cluster centre | 322313 | 14 | 168 |
| 2017 | Sokoto South | Sokoto | Nigeria | Cluster centre | 269807 | 14 | 144 |
| 2017 | Talata Mafara | Zamfara | Nigeria | Cluster centre | 304281 | 13 | 99 |
| 2017 | Tureta | Sokoto | Nigeria | Cluster centre | 94640 | 12 | 192 |
| 2017 | Wamako | Sokoto | Nigeria | Cluster centre | 248635 | 14 | 146 |
| 2017 | Zurmi | Zamfara | Nigeria | Cluster centre | 415512 | 13 | 89 |
| 2017 | Batsari | Katsina | Nigeria | Cluster | 289274 |  | 29 |
| 2017 | Binji | Sokoto | Nigeria | Cluster | 145382 | 16 | 27 |
| 2017 | Birnin Magaji/Kiyaw | Zamfara | Nigeria | Cluster | 252583 | 13 | 95 |
| 2017 | Bukkuyum | Zamfara | Nigeria | Cluster | 299268 | 17 | 33 |
| 2017 | Bungudu | Zamfara | Nigeria | Cluster | 364718 | 15 | 37 |
| 2017 | Goronyo | Sokoto | Nigeria | Cluster | 252340 | 14 | 58 |
| 2017 | Guidam-Roumdji |  | Niger | Cluster | 966372 |  | 31 |
| 2017 | Gummi | Zamfara | Nigeria | Cluster | 289236 | 14 | 45 |
| 2017 | Gusau | Zamfara | Nigeria | Cluster | 541825 | 13 | 93 |
| 2017 | Gwadabawa | Sokoto | Nigeria | Cluster | 320254 | 14 | 76 |
| 2017 | Jibia | Katsina | Nigeria | Cluster | 234971 | 10 | 43 |
| 2017 | Madarounfa |  | Niger | Cluster | 548890 | 16 | 115 |
| 2017 | Maru | Zamfara | Nigeria | Cluster | 412773 | 13 | 99 |
| 2017 | Sabon Birni | Sokoto | Nigeria | Cluster | 287366 |  | 11 |
| 2017 | Sandamu | Katsina | Nigeria | Cluster | 190037 |  | 2 |
| 2017 | Shagari | Sokoto | Nigeria | Cluster | 216512 |  | 7 |
| 2017 | Shinkafi | Zamfara | Nigeria | Cluster | 191820 | 12 | 236 |
| 2017 | Silame | Sokoto | Nigeria | Cluster | 144484 | 14 | 27 |
| 2017 | Tangaza | Sokoto | Nigeria | Cluster | 157599 | 18 | 26 |
| 2017 | Wurno | Sokoto | Nigeria | Cluster | 224671 | 16 | 81 |
| 2017 | Yabo | Sokoto | Nigeria | Cluster | 159202 | 15 | 46 |

**Table S4.** Vaccine use in reactive vaccination campaigns. Coverage data used in modelling cases in the absence of intervention. No data indicates districts where no specific coverage data were found other than a description of “full” or “partial” coverage.

| District | Country | Year | Population | Vaccine type | Doses | Coverage | No data |
| --- | --- | --- | --- | --- | --- | --- | --- |
| Dogon-Doutchi | Niger | 2015 | 385897 | ACW PS | 256622 | 95% | * |
| Dosso | Niger | 2015 | 519638 | ACW PS | 345559 | 95% | * |
| Filingue | Niger | 2015 | 594689 | ACW PS | 208141 | 50% | * |
| Illela | Niger | 2015 | 468833 | ACW PS | 311774 | 95% | * |
| Kollo | Niger | 2015 | 495869 | ACW PS | 329753 | 95% | * |
| Niamey I | Niger | 2015 | 224212 | ACW PS | 149101 | 95% | * |
| Niamey II | Niger | 2015 | 263583 | ACW PS | 175283 | 95% | * |
| Niamey III | Niger | 2015 | 174202 | ACW PS | 115844 | 95% | * |
| Niamey IV | Niger | 2015 | 293033 | ACW PS | 194867 | 95% | * |
| Niamey V | Niger | 2015 | 141209 | ACW PS | 93904 | 95% | * |
| Ouallam | Niger | 2015 | 419980 | ACYW conjugate | 279287 | 90% |  |
| Aliero | Nigeria | 2015 | 86835 | ACW PS | 30392 | 50% | * |
| Bunza | Nigeria | 2015 | 159869 | ACW PS | 66222 | 59% |  |
| Gudu | Nigeria | 2015 | 124663 | ACW PS | 82901 | 95% | * |
| Jega | Nigeria | 2015 | 254493 | ACW PS | 81975 | 46% |  |
| Maiyama | Nigeria | 2015 | 231241 | ACW PS | 21316 | 13% |  |
| Shagari | Nigeria | 2015 | 204083 | ACW PS | 51700 | 36% |  |
| Zuru | Nigeria | 2015 | 217896 | ACW PS | 144901 | 95% | * |
| Niamey II | Niger | 2016 | 272153 | ACW PS | 180982 | 95% | * |
| Goronyo | Nigeria | 2016 | 244991 | ACW PS | 162919 | 95% | * |
| Birni N'Konni | Niger | 2017 | 374442 | ACW PS | 131055 | 50% | * |
| Dioundiou | Niger | 2017 | 132321 | ACW PS | 87993 | 95% | * |
| Tibiri | Niger | 2017 | 325948 | ACW PS | 114082 | 50% | * |
| Kollo | Niger | 2017 | 560762 | ACW PS | 196267 | 50% | * |
| Madarounfa | Niger | 2017 | 548890 | ACW PS | 365012 | 95% | * |
| Niamey II | Niger | 2017 | 289437 | ACW PS | 192476 | 95% | * |
| Niamey III | Niger | 2017 | 191289 | ACW PS | 66951 | 50% | * |
| Niamey IV | Niger | 2017 | 321775 | ACW PS | 112621 | 50% | * |
| Anka | Nigeria | 2017 | 201197 | ACW PS | 29172 | 21% |  |
| Bakura | Nigeria | 2017 | 264300 | ACW PS | 19210 | 10% |  |
| Batagarawa | Nigeria | 2017 | 255495 | ACW PS | 6775 | 4% |  |
| Batsari | Nigeria | 2017 | 289274 | ACW PS | 12177 | 6% |  |
| Birnin Magaji/Kiyawa | Nigeria | 2017 | 252583 | ACW PS | 55254 | 31% |  |
| Bukkuyum | Nigeria | 2017 | 299268 | ACW PS | 199013 | 95% | * |
| Bungudu | Nigeria | 2017 | 364718 | ACW PS | 22165 | 9% |  |
| Damaturu | Nigeria | 2017 | 128498 | ACW PS | 38802 | 43% |  |
| Faskari | Nigeria | 2017 | 271358 | ACW PS | 6876 | 4% |  |
| Fika | Nigeria | 2017 | 199863 | ACW PS | 58811 | 42% |  |
| Fune | Nigeria | 2017 | 439100 | ACW PS | 14509 | 5% |  |
| Funtua | Nigeria | 2017 | 312243 | ACW PS | 5535 | 3% |  |
| Gada | Nigeria | 2017 | 343660 | ACW PS | 18786 | 8% |  |
| Gujba | Nigeria | 2017 | 189925 | ACW PS | 24676 | 19% |  |
| Gummi | Nigeria | 2017 | 289236 | ACW PS | 19070 | 9% |  |
| Gusau | Nigeria | 2017 | 541825 | ACW PS | 89030 | 23% |  |
| Jibia | Nigeria | 2017 | 234971 | ACW PS | 12545 | 8% |  |
| Katsina | Nigeria | 2017 | 440822 | ACW PS | 164 | 0% |  |
| Kaura Namoda | Nigeria | 2017 | 397878 | ACW PS | 125123 | 45% |  |
| Malumfashi | Nigeria | 2017 | 253204 | ACW PS | 1991 | 1% |  |
| Maradun | Nigeria | 2017 | 298163 | ACW PS | 14121 | 7% |  |
| Maru | Nigeria | 2017 | 412773 | ACW PS | 59443 | 21% |  |
| Shinkafi | Nigeria | 2017 | 191820 | ACW PS | 37904 | 28% |  |
| Talata Mafara | Nigeria | 2017 | 304281 | ACW PS | 42096 | 20% |  |
| Tsafe | Nigeria | 2017 | 376159 | ACW PS | 60874 | 23% |  |
| Zurmi | Nigeria | 2017 | 415512 | ACW PS | 23591 | 8% |  |
| Bodinga | Nigeria | 2017 | 242803 | C conjugate | 124450 | 73% |  |
| Dange-Shuni | Nigeria | 2017 | 269297 | C conjugate | 125037 | 66% |  |
| Goronyo | Nigeria | 2017 | 252340 | C conjugate | 36414 | 21% |  |
| Gwadabawa | Nigeria | 2017 | 320254 | C conjugate | 31485 | 14% |  |
| Isa | Nigeria | 2017 | 202241 | C conjugate | 42708 | 30% |  |
| Rabah | Nigeria | 2017 | 206479 | C conjugate | 97388 | 67% |  |
| Sokoto North | Nigeria | 2017 | 322313 | C conjugate | 154669 | 69% |  |
| Sokoto South | Nigeria | 2017 | 269807 | C conjugate | 133854 | 71% |  |
| Tureta | Nigeria | 2017 | 94640 | C conjugate | 47625 | 72% |  |

**Table S5.** Laboratory results in outbreak and non-outbreak periods. Data from Niger line list 2015-2017 and Nigeria line list 2015.

| Laboratory result | Non-outbreak period | | Outbreak period | |
| --- | --- | --- | --- | --- |
| No test reported | 5677 | 44% | 7538 | 47% |
| Test reported | 7257 | 56% | 8464 | 53% |
| Test result |  |  |  |  |
| Negative | 4636 | 64% | 5995 | 71% |
| *N. meningitidis* | 2175 | 30% | 2299 | 27% |
| *S. pneumoniae* | 355 | 5% | 139 | 2% |
| *H. influenzae* | 83 | 1% | 27 | <1% |
| Other bacteria | 8 | <1% | 4 | <1% |

**Figure S1.** Flowchart of work methodology. Raw data shown in green solid-lined boxes; modeled data in orange dotted-lined boxes; analyses shown in blue ovals.
